# Low-Frequency Repetitive Transcranial Magnetic Stimulation for Chronic Tension-Type Headache: A Randomized Controlled Study

**DOI:** 10.7759/cureus.34922

**Published:** 2023-02-13

**Authors:** Manju Rajain, Renu Bhatia, Manjari Tripathi, Nand Kumar, Rashmi Mathur

**Affiliations:** 1 Department of Physiology, National Institute of Medical Sciences and Research, Jaipur, IND; 2 Department of Physiology, All India Institute of Medical Sciences (AIIMS), New Delhi, IND; 3 Department of Neurology, All India Institute of Medical Sciences (AIIMS), New Delhi, IND; 4 Department of Psychiatry, All India Institute of Medical Sciences (AIIMS), New Delhi, IND

**Keywords:** sham therapy, repetitive transcranial magnetic stimulation, pain management, muscle overactivity, chronic tension-type headache, headache

## Abstract

Background: Nearly 1-3% of the population is affected by chronic tension-type headaches (CTTH). However, it is still difficult to treat owing to the lack of knowledge of the disease's pathophysiology. Available literature suggests a role for pericranial muscle activity and abnormal modulation of central pain. Repetitive transcranial magnetic stimulation (rTMS) therapy done at the dorsolateral prefrontal cortex (DLPFC) can help modulate pericranial muscle overactivity and central pain modulation in subjects with CTTH.

Aim: This randomized controlled study aimed to assess the effect of rTMS used in the low-frequency dorsolateral prefrontal cortex on pain and muscle activity in subjects with a chronic tension-type headache.

Materials and methods: The present randomized controlled clinical study was commenced in a health care center on 20 subjects with chronic tension-type headaches who were given either sham or low-frequency repetitive transcranial magnetic stimulation at the right dorsolateral prefrontal cortex. The therapy effect was evaluated statistically using Welch’s corrected t-test.

Results: The study results depicted that daily use of rTMS therapy for two weeks led to a considerable reduction in the intensity of the pain, the activity of pericranial muscles, and headache impact, along with an increase in the nociceptive excitability thresholds in subjects with CTTH, with p=0.001 compared to the sham group.

Conclusion: Considering its limitations, the present study depicts that rTMS is an efficacious management tool for reducing pain associated with CTTH and can serve as the cornerstone for additional investigations.

## Introduction

Tension-type headache (TTH) is a common type of headache that affects about 1-3% of the population globally. It presents itself as band-like pain felt by the subjects in the occipital, parietal, and frontal regions. Tension-type headaches can last from a few hours to many weeks. At least once in their lifetime, nearly 78% of adults experience a tension-type headache [1].

The chronic form of tension-type headache is known as chronic tension-type headache (CTTH), which presents as a disabling pain that largely affects the lifestyle of the affected subject and imparts a high socioeconomic burden for its management as it prevents subjects from carrying out their daily work activities. The pathogenesis is likely multifactorial and different for each TTH subtype. According to some research, the chronic variety of TTH may be significantly influenced by genetic and central factors, whereas episodic TTH may be more affected by peripheral and environmental factors, which makes it a challenging disease for treating personnel [2,3].

Analgesics and tricyclic antidepressants (TCAs) have been used as first-line pharmacological agents for managing the acute symptoms associated with tension-type headaches [4]. However, previous literature studies have reported that analgesics and TCAs have negligible or minimal efficacy in treating chronic tension-type headaches [3]. Considering their low efficacy, various non-pharmacological treatment strategies have been employed for treating chronic tension-type headaches. These non-pharmacologic management strategies, along with standard care, have proved to be popular and effective for treating chronic tension-type headaches [4].

One such treatment strategy is the use of repetitive transcranial magnetic stimulation (rTMS). The efficacy of rTMS use for treating chronic tension-type headaches and other chronic headaches has been established by various previous studies conducted in the literature. Despite having more side effects than selective serotonin reuptake inhibitors, tricyclic antidepressants are more effective at preventing migraines and tension-type headaches. Tricyclics appear to become more effective with time [4]. A recent study done by Mattoo et al. in 2019 reported that the literature evidence is limited for chronic tension-type headaches, warranting further studies to assess the efficacy of rTMS use as an adjunct to standard care for managing chronic tension-type headaches [5].

The aim of this study was to assess the efficacy of rTMS (1 Hz) applied to the right dorsolateral prefrontal cortex (DLPFC) in subjects with chronic tension-type headaches (CTTH). Also, the aim of this study was to assess whether the use of rTMS along with standard care has an additional benefit in clinical settings.

## Materials and methods

The present two-arm parallel, randomized clinical study was done at a tertiary health care center after clearance was provided by the Ethical Committee Board (Ref. No. IESC/T-81/28.02.2014, RT-15/30.03.2016) of the institute between February 2014 and March 2016, and informed consent was obtained in both written and verbal format from all the participants of the study. A visual analog scale (VAS) for pain was used to assess the effects of low-frequency rTMS, electromyography (EMG) for pericranial muscle activity, HIT-6 for headache impact, and nociceptive flexion reflex (NFR).

The study included 20 subjects with CTTH who attended the neurology and psychiatry departments of the institute. According to a study by Jackson et al. [4], inclusion criteria included subjects with CTTH who were diagnosed considering the criteria set by the International Headache Society for CTTH. The age range for study participants was 18-50 years. The exclusion criteria were subjects with a cardiac pacemaker, in need of analgesics three or more times daily, a history of seizures, an abnormal menstrual cycle, on hormonal therapy, lactating and pregnant females, subjects using illicit substances or morpho-mimetic drugs, muscle relaxants, and subjects practicing measures to relieve pain, including acupuncture, ferromagnetic implants used in the head and neck region, or yoga.

The power of the study was calculated at 80% using G-Power software and was normally distributed. Included were 20 participants who were randomly assigned to two groups of 10 each following the block-randomized sequence. In opaque and sealed envelopes, the allocations were concealed. After the final recruitment, each subject was assigned a code to maintain confidentiality concerning the therapy employed and personal details with a person not informed of the outcomes assessed. The details were also blinded to the statistician, assessor, and principal investigator.

Based on the resting motor threshold (RMT), the dose to be administered was estimated along with the stimulation intensity of the left abductor pollicis brevis. A 70-mm coil from Neurosoft (NeuroMS/D, Ivanovo, Russia) was placed in a figure-eight fashion anteroposteriorly on the scalp in the motor hotspot for the thumb muscles. Following the guidelines of the IFCN (International Fact-Checking Network), the recording site was localized. The minimum intensity needed to elicit the thumb twitch in >50% of the trials was the resting motor threshold. The optimal site for stimulation during the therapy was the DLPFC, which was marked and approximately 5 cm anterior to the motor hotspot for the thumb muscles. Five sessions were done every week for two consecutive weeks, making a total of 10 rTMS sessions. In Group I, each session lasted 25 minutes and used a fixed frequency of 1 Hz to stimulate at 110% of the resting motor threshold. The subjects from group II were managed by sham stimulation, which involves placing the coil perpendicular to the cranium without magnetic field penetration to the brain.

The outcomes assessed were pain intensity primarily, and the secondary outcomes were EMG (electromyography) parameters, nociceptive flexion reflex parameters, and disease impact. The assessment was done before and after the intervention within 24 hours. Subjects were called in the morning after the overnight fast and after proper rest.

The visual analog scale of 1988 by McCormack et al. [6] was used to evaluate pain experienced by study subjects; NFR was assessed using the Willer et al. [7] methods of 1977 and the Headache Impact Test-6 (HIT-6) by Bjorner et al. [8] in 1993. The sural nerve was stimulated with surface electrodes (Ag-AgCl) at the retromalleolus ankle side and was recorded from the biceps femoris (short head) with the surface electrodes. EMG data acquisition units from MP 30 and Biopac System, Inc., Santa Barbara, California, USA, and transcutaneous electrical stimulators from BLST-MA and Biopac System were used in the study.

Duration, latency, and threshold were assessed for NFR. EMG was done bilaterally for cervical and pericranial muscles, including the trapezius, the frontalis, and the temporalis muscles, and evaluated with surface electrodes on digital software (Student Laboratory System, BSL Pro V 3.6.7 model, Biopac System, Inc.). In maximal voluntary contractions, mental activity, and during rest, EMG was recorded, with each lasting for one minute. Time to fatigue and EMG amplitude were also assessed [5].

All data were checked for normality using D'Agostino and Pearson's omnibus normality test. The data gathered was analyzed using statistical tests and expressed in means and standard deviations. The test utilized to assess the role of low-frequency rTMS on the DLPFC concerning nociceptive excitability, muscle hardness, the impact of headache on daily life, and pain intensity in subjects with CTTH uses Welch’s correction test with a paired samples t-test and a t-test with 5% as the significance level. The statistical analysis was done with GraphPad Software, San Diego, CA, USA.

## Results

The participants in the study had the symptoms of headache for a mean of 6.35 ± 1.50 years and the range of 2 years to 8.5 years. All participants were refractory to the standard analgesics. The mean VAS scores were 5.3 ± 1.33 with a range of 3 to 7. Headache was affected bilaterally in 70% (n=14) of subjects and unilaterally in 30% (n=6) of subjects. There were 60% (n=12) females in the study, 75% (n=15) subjects residing in urban areas, 80% (n=16) subjects married, and 90% (n=18) subjects working full time. Standard drugs were continued for all the subjects (Table 1).

**Table 1:** Demographic details of the subjects VAS: visual analog scale, rTMS: repetitive transcranial magnetic stimulation

Gender	
Male	40% (n=8)
Female	60% (n=12)
Age	
rTMS	37.5±1.20
Sham	36.2±1.05
Duration	
Symptoms of headache	6.35±1.50 (2–8.5 years)
VAS	5.3±1.33 (3–7 years)
Location	
Unilateral	30% (n=6)
Bilateral	70% (n=14)

Before therapy initiation, subjects were divided into sham or rTMS groups. Parameters of EMG and NFR showed a non-significant difference between the two groups except for the increased activity of left and right trapezius muscles during mental activity. rTMS was well-tolerated by all the subjects, with no reported side effects during the complete therapy duration. All subjects completed all the sessions of the study.

The study results showed that subjects who received rTMS had significantly lower pain scores, which reduced from 6.00±1.53 to 1.10±1.13 post-therapy with p=0.001 compared to the sham group, where VAS reduced non-significantly from 5.60±1.15 to 5.55±1.19 post-therapy with p=0.5171 with a 95% confidence interval (CI) (Table 2).

**Table 2:** Comparison of VAS scores preoperatively and postoperatively in study rTMS: repetitive transcranial magnetic stimulation, VAS: visual analog scale

VAS scores	rTMS	Sham	P-value
Pre-therapy	6.00±1.53	5.60±1.15	0.5171
Post-therapy	1.10±1.13	5.55±1.19	0.001
P-value (pre- vs post-therapy)	0.001	0.937	

Similarly, a higher and significant reduction in HIT-6 scores was seen postoperatively for the rTMS group, from 63.00±5.68 to 42.90±7.09 with p=0.001 compared to the sham groups, where HIT-6 scores reduced non-significantly from 59.10±7.84 to 57.00±7.54 postoperatively with p=0.2189 (Table 3).

**Table 3:** Comparison of HIT-6 scores preoperatively and postoperatively in study subjects HIT-6: headache impact test-6, rTMS: repetitive transcranial magnetic stimulation

HIT-6	rTMS	Sham	P-value (real vs sham)
Pre-therapy	63.00±5.68	59.10±7.84	0.2189
Post-therapy	42.90±7.09	57.00±7.54	0.001
P-value (pre- vs post-therapy)	0.001	0.146	

The threshold for NFR and rTMS increased significantly postoperatively, from 19.80±6.65 to 32.67±5.96, with p = 0.001. A similar significant increase was seen for latency from 104.10±13.02 to 140.15±12.77 with p=0.001. For the duration, a non-significant increase was seen from 41.44±4.03 to 41.83±4.32 (p=0.103). In the sham group, a non-significant increase was seen for threshold, latency, and duration with respective p-values of 0.479, 0.966, and 0.104. Threshold and frequency preoperatively were comparable between the rTMS and sham groups with p=0.9380 and 0.7493, respectively, whereas latency was significantly higher for rTMS in comparison to sham with p=0.001. Postoperatively, on the inter-group comparison, threshold and latency were significantly higher for the rTMS group in comparison to the sham group with p=0.001 for both, and the duration was higher for the sham group with p=0.001 (Table 4).

**Table 4:** Inter-group and intra-group comparison of HIT-6 scores in study subjects NFR: nociceptive flexion reflex, rTMS: repetitive transcranial magnetic stimulation, HIT-6: headache impact test-6

NFR		rTMS	Sham	P-value (real vs sham)
Threshold	Pre-therapy	19.80±6.65	20.00±4.49	0.9380
	Post-therapy	32.67±5.96	20.24±4.27	0.001
	P-value (pre- vs post-therapy)	0.001	0.479	
Latency	Pre-therapy	104.10±13.02	101.30±11.77	0.0001
	Post-therapy	140.15±12.77	101.35±10.63	0.001
	P-value (pre- vs post-therapy)	0.001	0.966	
Duration	Pre-therapy	41.44±4.03	42.07±4.36	0.7493
	Post-therapy	41.83±4.32	42.52±4.63	0.001
	P-value (pre- vs post-therapy)	0.103	0.104	

On the EMG, the muscle activities were assessed for both rTMS and sham. It was seen that following rTMS, the muscle activity for left and right temporalis decreased significantly from 80.20±29.17 to 33.05±9.96 and 77.96±15.60 to 39.73±8.94 (p<0.0001), whereas following sham, a non-significant reduction was seen from 71.66±21.51 to 70.55±21.16 (p=0.0742) and from 73.60±23.45 to 72.03±22.93 (p=0.074). After rTMS, muscle activity in the left and right frontalis decreased from 74.62±20.61 to 37.04±9.83 (p<0.0001) and 74.54±23.88 to 29.09±8.44 (p=0.0001), respectively. After sham, a non-significant reduction was seen from 78.55±20.32 to 78.06±19.87 (p=0.237) and from 69.59±18.27 to 69.42±18.45 (p=0.9837) for the left and right frontalis muscles, respectively. For the left trapezius muscle, following rTMS, a significant reduction was seen from 97.10±12.28 to 65.78±13.48 (p-0.001) and a non-significant activity reduction for the right trapezius from 77.05±19.78 to 52.49±17.52 (p=0.103). After the sham, a non-significant activity reduction was seen for the left trapezius from 95.20±12.15 to 94.81±12.04 (p<0.0001) and a non-significant increase from 80.54±17.67 to 81.20±16.89 (p=0.267) for the right trapezius muscle was noted. Muscle activities were comparable between the two groups pre-operatively with p=0.4658, 0.6304, 0.6727, 0.7320, 0.6823, and 0.2457, respectively, for the left temporalis, right trapezius, right temporalis, left frontalis, right frontalis, and left trapezius. Postoperatively, inter-group values were significant for all muscle activities, with lesser activity in the rTMS group (Table 5).

**Table 5:** Muscle activity on EMG after rTMS and sham rTMS: repetitive transcranial magnetic stimulation, EMG: electromyography

		rTMS	Sham	P-value (real vs sham)
Left temporalis	Pre-therapy	80.20±29.17	71.66±21.51	0.4658
	Post-therapy	33.05±9.96	70.55±21.16	0.0001
	P-value (pre- vs post-therapy)	0.001	0.0742	
Right temporalis	Pre-therapy	77.96±15.60	73.60±23.45	0.6304
	Post-therapy	39.73±8.94	72.03±22.93	0.001
	P-value (pre- vs post-therapy)	0.001	0.074	
Left frontalis	Pre-therapy	74.62±20.61	78.55±20.32	0.6727
	Post-therapy	37.04±9.83	78.06±19.87	0.0001
	P-value (pre- vs post-therapy)	0.001	0.237	
Right frontalis	Pre-therapy	74.54±23.88	69.59±18.27	0.7320
	Post-therapy	29.09±8.44	69.42±18.45	0.0001
	P-value (pre- vs post-therapy)	0.0001	0.9837	
Left trapezius	Pre-therapy	97.10±12.28	95.20±12.15	0.6823
	Post-therapy	65.78±13.48	94.81±12.04	0.0001
	P-value (pre- vs post-therapy)	0.001	0.174	
Right trapezius	Pre-therapy	77.05±19.78	80.54±17.67	0.2457
	Post-therapy	52.49±17.52	81.20±16.89	0.0001
	P-value (pre- vs post-therapy)	0.103	0.267	

## Discussion

The present randomized controlled exploratory study works to fill the gap by assessing the role of low-frequency rTMS on DLPFC concerning nociceptive excitability, muscle hardness, the impact of headaches on daily life, and pain intensity in subjects with CTTH. The study had 60% (n = 12) females, 75% (n = 15) subjects residing in urban areas, 80% (n = 16) subjects married, and 90% (n = 18) subjects working full time. The participants in the study had symptoms of headache for a mean of 6.35±1.50 years and a range of 2 years to 8.5 years, with an average lasting >4 hours with moderate intensity. Headache was affected bilaterally in 70% (n = 14) of subjects and unilaterally in 30% (n = 6). All the participants completed the study. These characteristics were comparable to previous work by Passard et al. [9] in 2007 and Pleger et al. [10] in 2004, where authors assessed subjects with demographic data comparable to the present study.

Following the application of sham and rTMS, it was seen that subjects who received rTMS had significantly lower pain scores, which reduced from 6.00±1.53 to 1.10±1.13 post-therapy with p<0.0001 compared to the sham group, where VAS reduced non-significantly from 5.60±1.15 to 5.55±1.19 post-therapy with p = 0.5171 (Table 2). Similarly, the rTMS group saw a higher and more significant reduction in HIT-6 scores postoperatively, from 63.005.68 to 42.907.09 with p < 0.0001 compared to the sham groups, where HIT-6 scores decreased non-significantly from 59.107.84 to 57.007.54 with p = 0.2189. These results were consistent with the 2019 study by Mattoo et al. [5], which was a pilot study. This further supports the fact that the adjuvant use of rTMS to conventional standard care in subjects with CTTH has added advantages concerning nociceptive excitability, muscle overactivity, daily life interference, and pain intensity.

The study results depict a significant reduction in nociceptive excitability following rTMS therapy. In cases of chronic migraine, the pain becomes largely unrelated to the triggers that initially brought it on and is frequently accompanied by "limbic" symptoms like sleep problems, exhaustion, impaired memory and concentration, and decreased libido. Therefore, it is conceivable that DLPFC activation could reduce or reset fronto-limbic dysfunction, leading to an improvement in the clinical situation, which was also confirmed by Lefaucheur et al. [11] in 2008 and Brighina et al. [12] in 2011. However, the exact mechanism and neuronal circuits utilized by rTMS are still unclear, as reported by Kumar et al. [13] in 2021.

The results of this study are promising concerning the use of rTMS in subjects with chronic tension-type headaches. However, their generalized applicability is limited owing to the use of the manual method for DLPFC localization, which was done considering the anatomical landmarks, which can have individual variations, which were also confirmed by Pommier et al. [14] in 2017. A previous literature study by Sevel et al. [15] in 2016 suggested that inaccurate therapy site localization can compromise the rTMS efficacy in CTTH. However, it is still unclear whether accurate localization can increase analgesic efficacy. Hence, further longitudinal studies are needed to comparatively evaluate the efficacy of rTMS with neural components. The study had many strengths, including the completion of all the sessions by all participants, minimal bias due to blinding, reliable results and outcomes, and an apt study design.

rTMS was also found to be a safer technique, with no side effects reported by the study participants. Another study by Teo et al. [16] in 2014 reported mild discomfort in their subjects while assessing the RMT, which was comparable in the sham and rTMS groups. The findings of this exploratory study further need to be established by a randomized controlled study with larger sample numbers and a longer evaluation duration. Also, the efficacy, sustainability, and safety of low-frequency rTMS stimulation for CTTH need to be confirmed by future studies.

The limitation of the study is that the possibility of a greater sham effect with the rTMS group cannot be ruled out with a smaller sample size.

## Conclusions

The current study suggests that adding rTMS to standard treatment has a superior impact on CTTH subjects' head and neck muscle activities, nociceptive excitability, interference with daily life, and pain intensity. The study suggests additional research for the improvement of rTMS-based therapies in subjects with CTTH.
